# The Analysis of the Physicochemical Properties of Benzocaine Polymorphs

**DOI:** 10.3390/molecules23071737

**Published:** 2018-07-16

**Authors:** Magdalena Paczkowska, Gabriela Wiergowska, Andrzej Miklaszewski, Anna Krause, Magdalena Mroczkowka, Przemysław Zalewski, Judyta Cielecka-Piontek

**Affiliations:** 1 Department of Pharmacognosy, Faculty of Pharmacy, Poznan University of Medical Sciences, 60-781 Poznan, Poland; mpaczkowska@ump.edu.pl (M.P.); gabriela.wiergowska@pozlab.pl (G.W.); magdalena.urbaniak1989@gmail.com (M.M.); pzalewski@ump.edu.pl (P.Z.); 2 Pozlab sp.z.o.o., 60-775 Poznan, Poland; anna.krause@pozlab.pl; 3 Institute of Materials Science and Engineering, Poznan University of Technology, 60-965 Poznan, Poland; andrzej.miklaszewski@put.poznan.pl

**Keywords:** structural polymorphism, physicochemical properties, benzocaine, dissolution, permeability

## Abstract

The study was a pioneering attempt to assess the influence of the structural polymorphism (forms I, II, III) of benzocaine on its solubility, apparent solubility, and chemical stability, which are vital parameters for preformulation and formulation work. The impact of differences in the solubility of selected polymorphs of benzocaine on their permeability through artificial biological membranes (PAMPA system) was evaluated. The polymorphs of benzocaine were obtained by means of techniques commonly used for the preparation of various pharmaceutical dosage forms: ball milling, micro milling, and cryogenic grinding, which allowed for the appearance or preservation of form III, the initial conformation of benzocaine. Ball milling resulted in the conversion of form III to I, whereas micro milling yielded form II. As a result of cryogenic grinding, form III of benzocaine was preserved. The identification of all polymorphic forms of benzocaine was confirmed via X-ray powder diffraction (PXRD) supported by FT-IR spectroscopy coupled with density functional theory (DFT) calculations. The differences in solubility, dissolution, and permeability through artificial biological membranes resulting from the polymorphic forms of benzocaine were established by using chromatographic determinations. Accelerated stability tests indicated that all polymorphic forms were chemically stable at a required level.

## 1. Introduction

Benzocaine, which has been used in medicine for over a century [1], is a local anesthetic that acts by inhibiting voltage-dependent sodium channels [2]. It is present in many topical preparations for local application, such as dental sprays, throat anesthesia lozenges, skin gels and powders, ear drops, and condom desensitizers. Benzocaine is often administered with other active pharmaceutical ingredients (APIs) and excipients. This variety of dosage forms necessitates a large number of techniques for processing benzocaine. The most common of them are ball milling, micro milling, and cryogenic grinding. Since structural polymorphism is frequently observed in APIs, each of those processes may lead to the formation or change of particular polymorphic forms of APIs exhibiting different physicochemical properties (solubility, dissolution profile, chemical stability, and permeability through biological barriers) related to bioavailability, which determines their therapeutic safety and effectiveness [3,4,5,6,7,8]. At present, benzocaine is known to exist in three polymorphic forms. Form I, formerly form β, is a monoclinic P 21/c polymorph (Z = 4): CCDC codes: QQQAXG02 and QQQAXG04) [9]. Form II, previously α, is an orthorhombic P 21, 21, 21 polymorph (Z = 4): CCDC code: QQQAXG01) [10]. Form III is a monoclinic P 21 polymorph (Z = 8): CCDC code: QQQAXG03) [11]. E.J. Chan reported that conversion from form II to form III of benzocaine resulted in a twinned structure with a new cell having the b axis doubled and a monoclinic P112_1_ structure [10]. The difference between forms I and II is connected with flat ribbons of structure that propagate along the a direction. These consist of two symmetry-related halves [10]. At a low temperature, form II of benzocaine becomes form III [12]. A similar phase transition has not been observed for form I. Considering the melting point values, form II of benzocaine is the most stable of the three solid phases under ambient conditions. Due to the fact that benzocaine is classified as a poorly soluble anesthetic, it is vital to evaluate the influence of its polymorphic forms on physicochemical properties important for the preparation of various pharmaceutical dosage forms. To the best of our knowledge, there have been no reports on how the structural polymorphism of benzocaine affects its solubility, dissolution profile, chemical stability, and permeability through biological barriers. So far studies have concentrated on characterization of the polymorphic forms of benzocaine with the use of X-ray crystallography [13]. An unstable ester bond (ester of *p*-aminobenzoic acid) in the structure of benzocaine has stimulated interest exclusively in its chemical stability with no attention given to the impact of specified polymorphic forms [14,15]. Accelerated stability tests conducted according to ICH regulations (The International Council for Harmonisation of Technical Requirements for Pharmaceuticals for Human Use) indicated that benzocaine of an unidentified crystalline form was stable for 6 months in the air of increased relative humidity (RH 50%) and temperature (T = 333 K). Compatibility studies on benzocaine and selected excipients proved that its chemical stability depended on the presence of certain excipients, which were defined as an additional source of humidity.

The purpose of the present study was to evaluate the effect of techniques employed for the development of new pharmaceutical dosage forms on the formation of different benzocaine polymorphs and to assess the influence of selected polymorphic forms of the compound on its physicochemical properties, such as solubility, apparent solubility, and chemical stability. Their permeability through biological membranes was also assessed to establish the indirect effect of differences in the solubility of the polymorphic forms. 

## 2. Results and Discussion

The first part of the work involved preparing the polymorphic forms of benzocaine by using ball milling, micro milling, and cryogenic grinding. Their identification was conducted with methods based on X-ray powder diffraction (PXRD) supported by FT-IR spectroscopy coupled with DFT calculations.

**Figure 1:**
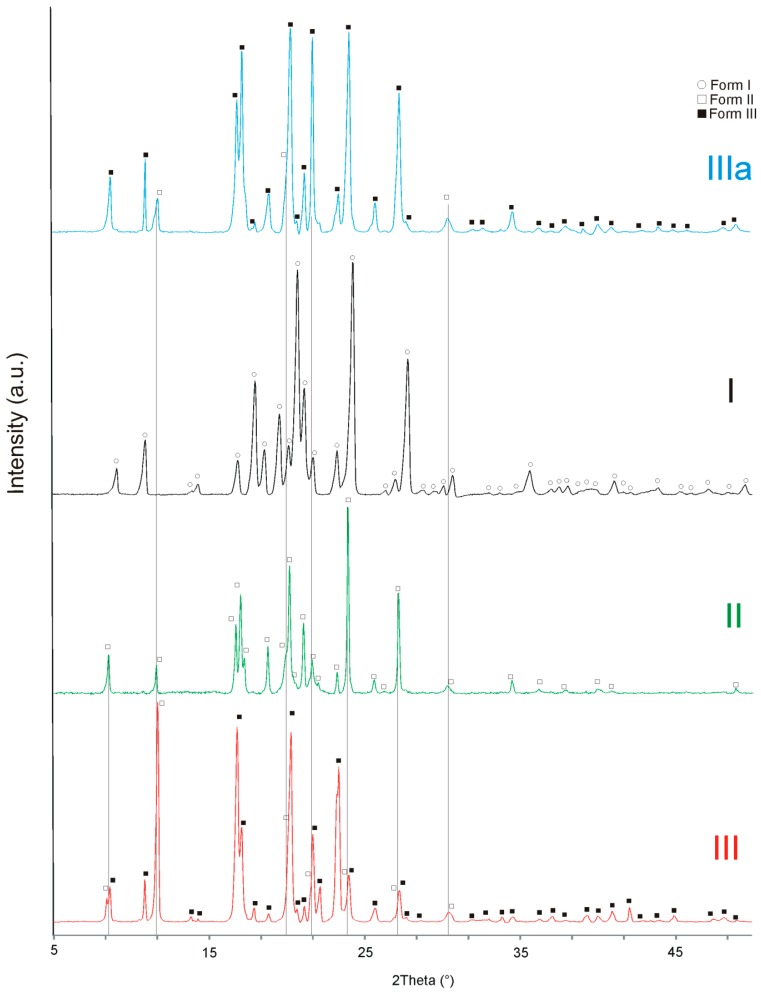
PXRD diffractograms of polymorphic forms of benzocaine.

Structural analysis was conducted on prepared samples and is depicted in Figure 1 within the spectra indication for predominant phase occurrence. As for the micro-milled samples provided by Galex (Murska Sobota, Slovenia) stabile form I (Z = 4): CCDC code: QQQAXG02 was confirmed. For the initial benzocaine sample provided by Pharma Cosmetics (Krakow, Poland), PXRD data confirmed the polymorphic form III (Z = 8): CCDC code: QQQAXG03), according to the reference data [11], with some residual amount of form II P212121 (Z = 4): CCDC code: QQQAXG01 (Figure 1). A tendency for the occurrence of forms III and II as the favored ones under such experimental conditions has also been reported [12]. After the cryogenic grinding of the initial benzocaine samples, form III was maintained for benzocaine, although minor changes in the spectrum were noticed (form IIIa). It is possible to suggest that form IIIa is a mixture of III and I forms. As may be observed from the comparison of the diffractograms of the initial samples of benzocaine with the diffractograms of benzocaine after cryogenic grinding, the reflections of the second phase were considerably reduced. This could be explained by the processing at a low temperature during cryogenic grinding and then the achievement of room temperature. On top of the fading reflections of form II, the broadening reflections of form III were noticed. This structural dependence may be explained by a decrease in the material grain size caused by the milling. After the ball milling of the initial benzocaine samples, the transformation of form III with a residual amount of form II to form I is observed. One way to explain the structural response of the material under the processing conditions is that the milling probably increased the temperature locally and transformed the system during repeatable collisions between the powder and the milling ball.

**Figure 2:**
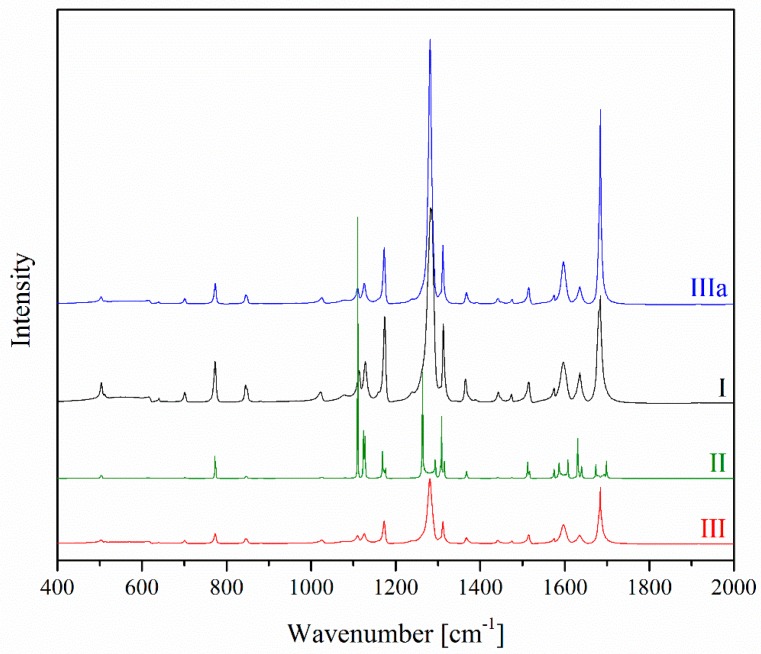
FT–IR spectra of polymorphic forms of benzocaine.

The identification of characteristic bands within the FT–IR spectra of the three polymorphic forms of benzocaine was supported by comparison with theoretical spectra obtained through applying the density functional theory with the B3LYP (Becke, 3-parameter, Lee-Yang-Parr) hybrid functional and the 6–31 G (d,p) basis set (Figure 2). The calculated vibrational frequencies were scaled in order to improve agreement with the experimental values. The assignment of a vibrational model to particular domains in a benzocaine molecule relative to the FT–IR spectra is summarized in Table 1. The most characteristic bands associated with the vibration of bonds present in the specified forms of benzocaine were located between 500 and 2000 cm^−1^. It was possible to identify polymorphic form III due to the presence of two bands that were more intense in comparison with the FT–IR spectra of other polymorphic forms in the range 1280–1300 cm^−1^ associated with the stretching vibration of the C–O and C–C bonds and the wagging vibration of the C–H bonds, and a band at 1682 cm^−1^ corresponding to the stretching vibration of the C=O bond. In contrast to the FT-IR spectra of forms I and II, for form III a characteristic change was the decreasing intensity of the band at 773 cm^−1^ linked with the out-of-plane bending vibration of the O–C=O bond. It was also observed that additional bands appeared in the range 1350–1500 cm^−1^, associated with the scissoring and rocking vibrations of the C–H bond. Table 1 lists a selection of the characteristic bands of benzocaine. The dark lines contain the bands and the corresponding vibrations important for differentiating the polymorphic forms of benzocaine. 

Finally, it was confirmed that the polymorphic form III (monoclinic P 21—Z = 8): CCDC code: QQQAXG03) of benzocaine was represented by the initial sample of benzocaine. Depending on the pharmaceutical technology procedure applied, different polymorphic forms of benzocaine appeared. Ball milling resulted in the conversion of form III to I (orthorhombic P 21, 21, 21 polymorph, Z = 4 CCDC code: QQQAXG02 and QQQAXG04) whereas micro milling yielded form II (monoclinic P 21/c polymorph, Z = 4 CCDC code: QQQAXG01).

Cryogrinding did not cause any change in the polymorphic form III of benzocaine. According to the literature, this may be a consequence of the fact that the techniques applied require low temperatures [12]. In summary, the study indicated that it was possible to obtain solids containing any of the three polymorphic forms of benzocaine through ball milling, cryogrinding, and micro milling. It should be stressed at this stage of research that the significant physical instability of benzocaine resulting in an easy transition between its polymorphic forms was not coupled with their chemical instability despite the presence of a labile ester bond in a benzocaine molecule. It was also demonstrated, on the basis of chromatographic analysis, that the main degradation product of benzocaine (*p*-aminobenzoic acid) was not formed under the conditions of the technological processes applied in this work.

**Figure 3:**
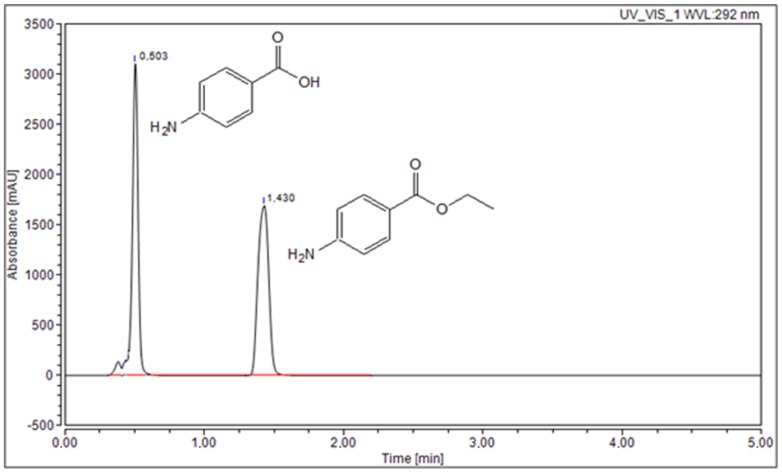
HPLC chromatogram of benzocaine in the presence of its main impurity (*p*-aminobenzoic acid).

The second part of the study focused on the physicochemical properties (solubility, apparent solubility profiles, chemical stability, and permeability through artificial biological membranes) of the three polymorphic forms of benzocaine. The physicochemical properties of the polymorphic forms of benzocaine were evaluated with the use of an HPLC-DAD method developed for the determination of benzocaine in the presence of its main impurity, *p*-aminobenzoic acid (Figure 3). The HPLC-DAD method was validated for the determination of benzocaine in methanol and a phosphate buffer (pH~7.2), according to ICH guidelines [16].

As a poorly soluble anesthetic, benzocaine dissolves better in methanol, which is often used in the preparation of the compound. The solubility studies of benzocaine were conducted in a phosphate buffer (pH ≈ 7.2) because the majority of pharmaceutical forms are designed to release benzocaine into an acceptor medium of a similar pH value (e.g., saliva or skin). Irrespective of the solvent type, differences were observed in the solubility of the three polymorphic forms of benzocaine, with forms I and II more soluble than form III (Table 2, Table 3 and Table 4). That may be explained by the presence of impurities confirmed in PXRD studies and attributed to the process of the benzocaine sample preparation.

**Figure 4:**
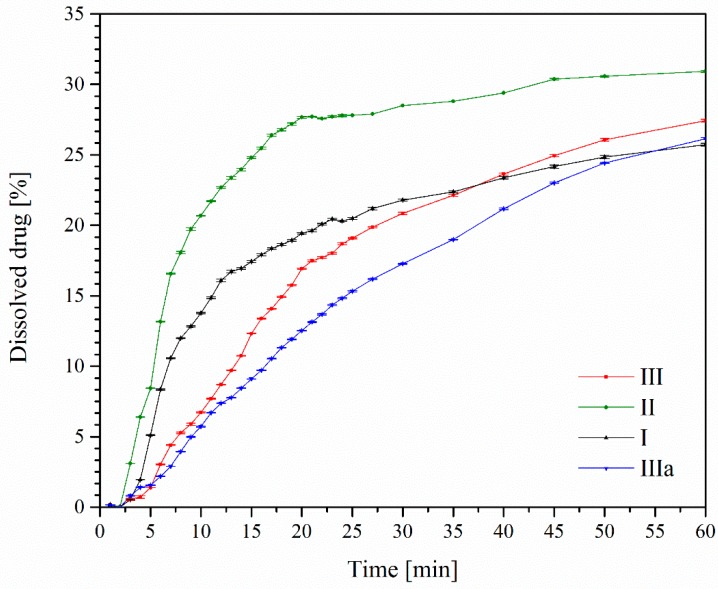
Apparent solubility profiles of benzocaine in phosphate buffer (pH ≈ 7.2).

Considering the action mechanism and the application site of benzocaine, its apparent solubility in a phosphate buffer of pH ≈ 7.2 was also assessed. By studying the apparent solubility, it is possible to determine the rate at which selected forms of APIs dissolve. In our study, apparent solubility was determined for the three polymorphic forms of benzocaine. The greatest difference in their apparent solubility was observed 10 min after the start of the experiment. The polymorphic form II of benzocaine dissolved more than four times as fast as form I, whereas form III ranked between those forms in that respect. The shapes of dissolution profile curves for forms I and II were comparable, while form III differed in its dynamics of apparent solubility. Differences were registered within sections of plateau curves indicating that form II showed the best apparent solubility (Figure 4).

**Figure 5:**
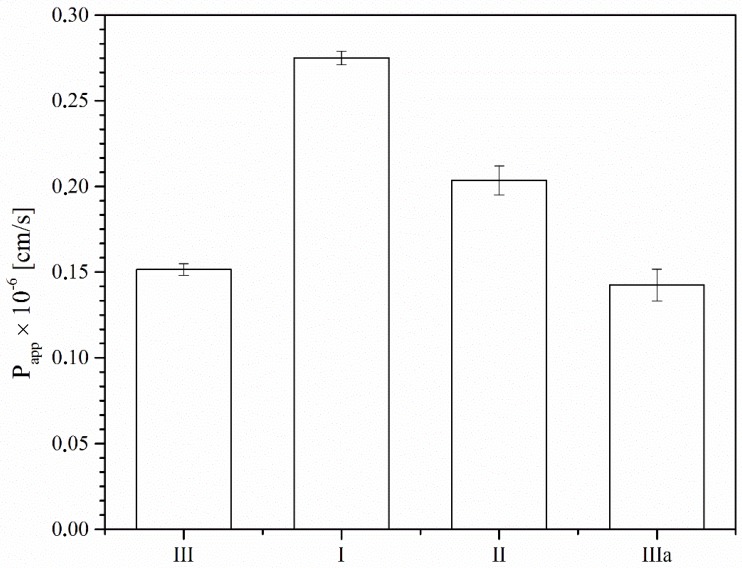
Apparent permeability coefficient (*P_app_*) for polymorphic forms of benzocaine.

Taking into consideration differences in apparent solubility for the three polymorphic forms of benzocaine, the study was continued to investigate the compound’s permeability through systems of biological membranes. As the PAMPA model is based on the assessment of changes in the concentrations of analytes in acceptor and donor fluids resulting from diffusion, changes in apparent solubility determine changes in permeability as a function of time. It should be stressed that since benzocaine, as a poorly soluble anesthetic, may demonstrate low permeability, any changes in this parameter may affect the compound’s activity profile. As expected, permeability studies of the three polymorphic forms of benzocaine by using the PAMPA system confirmed that the apparent permeability coefficients for all forms were less than 1 × 10^−6^ cm s^−1^, classifying them as low-permeability APIs [17,18,19]. Form II, which exhibited the greatest permeability (0.26 × 10^−6^ cm s^−1^), permeated through the system of biological membranes used in the study at a rate twice as high as that observed for form III (Figure 5). Those differences correlated positively with differences in solubility and dissolution profiles.

**Figure 6:**
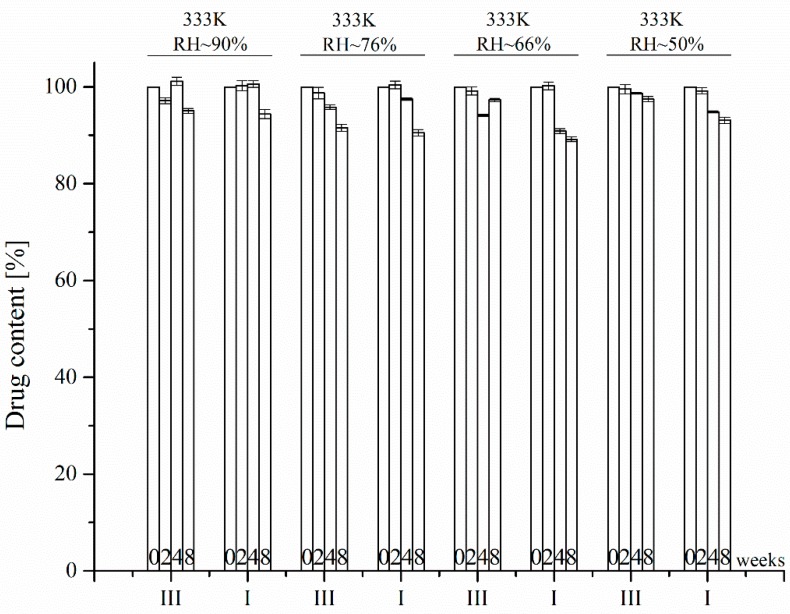
Benzocaine degradation results.

The last stage of research into the effect of polymorphism on the physicochemical properties of APIs involved investigation of the chemical stability of the previously mentioned polymorphic forms of benzocaine. The presence of an ester bond in benzocaine puts it in a class of chemically labile APIs characterized by a potential susceptibility to degradation, especially under the influence of water molecules. Previous studies have not indicated any degradation of benzocaine in the solid state when stored for 6 months at 298 K and RH = 60%, or under accelerated aging conditions at 313 K and RH = 75%. However, in the presence of certain excipients, benzocaine was prone to degradation, which was linked to their humidifying effect [14,15]. It was therefore decided that the present study would investigate accelerated degradation of the polymorphic forms of benzocaine exposed to stronger affecting factors, such as the RH range 50–90% at T = 333 K, in order to assess differences in their chemical stability resulting from increased humidity. Particular attention was directed to the degradation path to determine whether it was oriented toward the formation of *p*-aminobenzoic acid as the main degradation product of a probable hydrolysis of the ester bond in benzocaine. Accelerated degradation experiments did not indicate any degradation of the polymorphic forms of benzocaine. Similarly, an increase in the temperature (T = 383 K) in dry air did not cause any degradation related to the polymorphic forms I, II, III, and IIIa of benzocaine (Figure 6).

## 3. Materials and Methods

### 3.1. Materials

The initial samples of benzocaine (form III) (purity > 98%) was supplied by Pharma Cosmetic K.M. Adamowicz Sp. z.o.o. (Krakow, Poland). Acetonitrile of an HPLC grade was supplied by Merck KGaA (Darmstadt, Germany) and formic acid (100%) by Avantor Performance Materials (Gliwice, Poland). High-quality pure water was prepared using an Exil SA 67120 Millipore purification system (Molsheim, France). Hydrochloric acid, sodium hydroxide solution, hydrogen peroxide, potassium dihydrogen phosphate, potassium bromide, and all other chemicals were obtained from Avantor Performance Materials (Gliwice, Poland).

Benzocaine (form I) was obtained from GaleX dd. (Murska Sobota, Slovenia), and according to the specification, had been subject to micro milling. 

### 3.2. Preparation of Polymorphic forms of Benzocaine

Initial samples of benzocaine (form III) were subjected to ball milling and cryogenic milling. The ball milling of benzocaine was performed by using a Retsch MM 400 mill (Retsch, Katowice, Poland). A steel jar (50 mL) was filled with a sample of benzocaine (2 g) and 1 steel ball (30 mm in diameter). The rotation speed was set to 1800 rpm with a frequency of 30 Hz. The total time of milling at room temperature was 60 min. After milling, the benzocaine samples were stored at room temperature. The cryogenic milling of benzocaine was carried out by using a Retsch MM 400 mill. The total mass of benzocaine was 2 g. The samples were placed in a stainless-steel vessel and then inserted in liquid nitrogen. After 10 min, the samples were subjected to milling. The milling was set at an impact frequency of 10 Hz. The effective milling time was 60 min. After cryogenic milling, the benzocaine samples were stored at room temperature.

### 3.3. Identification of Polymorphic Forms of Benzocaine

The identification of all polymorphic forms of benzocaine was confirmed by an X-ray powder diffraction (PXRD) as the main technique and FT-IR spectroscopy coupled with DFT calculations as supporting methods.

### 3.4. X-ray Powder Diffraction (PXRD)

To analyze the initial and processed conformation of benzocaine, an X-ray powder diffraction (PXRD) technique was used. The diffractograms were collected by a PANalytical Empyrean system (Malvern Panalytical, Malvern, UK) with a Cu lamp (1.54056 Å). The measurements were carried out at a scanning range between 3° and 50° at 2θ range using a step size of 0.017° and a step time of 15 s/step with source parameters of 45 kV and 40 mA. For plot analysis, the backgrounds were automatically corrected using WinPLOTR software and then subtracted from spectra by spline interpolation.

### 3.5. Theoretical Analysis

Theoretical FT-IR spectra of benzocaine were obtained with the use of density functional theory calculations using Becke’s three-parameter hybrid functional (B3LYP) with 6–31++ (d,p) basis set. All ab initio calculations were carried out using the Gaussian 03 package [20]. Although the calculations of theoretical spectra of benzocaine were conducted for an isolated molecule in a gas state, differences between experimental and scaled wavenumbers values were small, therefore a detailed interpretation of FT-IR spectra of benzocaine was possible.

### 3.6. Fourier Transform Infrared Spectroscopy

The polymorphs of benzocaine were obtained separately with IR grade KBr at the ratio 1:100, and corresponding pellets were prepared by applying 8 metric ton of pressure in a hydraulic press. The vibrational infrared spectra were recorded between 400 and 2000 cm^−1^ with an FT-IR Bruker Equinox 55 spectrometer (Bruker, Bremen, Germany) equipped with a Bruker Hyperion 1000 microscope (Bruker, Bremen, Germany). In order to analyze changes in positions and intensity in experimental FT-IR spectra for the polymorphs of benzocaine, quantum chemical calculations based on DFT for benzocaine were performed with the use of the Gaussian 09 package [20].

### 3.7. Studies of Physicochemical Properties of Polymorphic Forms of Benzocaine

The evaluation of the effect of the structural polymorphism of benzocaine on its solubility, stability, dissolution, and permeability through an artificial membrane simulating gastrointestinal permeation was conducted by using chromatographic determinations. For this purpose, an HPLC-DAD method was developed and validated according to ICH Q2 recommendations with regard to the determination of changes in benzocaine concentrations during the said studies. The HPLC system (DionexThermoline Fisher Scientific, Waltham, MA, USA) was equipped with a high-pressure pump (UltiMate 3000), an autosampler (UltiMate 3000) and a DAD detector (UltiMate 3000). For data processing and acquisition, Chromeleon software version 7.0 from Dionex Thermoline Fisher Scientific (Waltham, MA, USA) was used. The separation of benzocaine and its main impurity—*p*-aminobenzoic acid—was achieved on a Kinetex C18 (100 × 2.10 mm, 2.6 µm) column (Phenomenex, Torrance, CA, USA) using a mobile phase composed of acetonitrile–0.1% formic acid (30:70 *v*/*v*) at a flow rate of 0.5 mL min^−1^. The injection volume was 5.0 µL and the wavelength of detection was controlled at 292 nm.

### 3.8. Solubility Studies

The phase-solubility profiles of the three polymorphic forms of benzocaine were determined according to the method of Higuchi and Connors [21], and were performed by using a Thermo Scientific™ MaxQ™ 4450 Benchtop Orbital Shaker (Thermo Scientific, Waltham, MA, USA). To 25.0 mL flasks, containing 10.0 mL methanol or phosphate buffer (pH ~ 7.2), increasing amounts of selected polymorphic forms of benzocaine were added until supersaturated solvents were received. The flasks were shaken at room temperature (methanol solvents) or 310 K (buffer solvents) for 5 h at a frequency of 400 rpm. The so-obtained solutions were filtered through a 0.45 µm filter.

### 3.9. Apparent Solubility Profiles

Apparent solubility (solubility rate) studies of the three polymorphic forms of benzocaine were performed by using an Agilent 708-DS Dissolution Apparatus (Agilent, Santa Clara, CA, USA). The standard paddle method was used at 310 ± 0.5 K with a stirring speed of 50 rpm. The polymorphic forms of benzocaine (I, II, and III) weighted into gelatin capsules were placed in a capsule sinker in order to prevent the capsules from floating on the surface of the liquid. The so-obtained samples were placed in 500 mL media of phosphate buffer (pH ≈ 7.2). The dissolved samples (5.0 mL) were collected at specified time intervals with the replacement of an equal volume of temperature-equilibrated media, filtered through a 0.45 μm membrane filter, and benzocaine concentrations were determined by using the HPLC-DAD method. All apparent solubility profiles were compared with an independent mathematical approach model using a similarity factor. 

### 3.10. Permeability Studies

Studies of the permeability the polymorphic forms of benzocaine through biological membranes were conducted by using a parallel artificial membrane permeability assay (PAMPA) evolution from Pion, Inc. (Pion Inc., Billerica, MA, USA). The PAMPA consisted of a 96-well microfilter plate and a 96-well filter plate, so that each sample was able to diffuse into two chambers, with a donor at the bottom and an acceptor at the top, separated by a 120-μm-thick microfilter disc coated with a 20% (*w*/*v*) dodecane solution of a lecithin mixture (Pion, Inc.). Samples of the polymorphic forms of benzocaine (20.0 mg) were dissolved in water, prepared in a different 96-well filter plate and added to the donor compartments. The donor solution was adjusted to pH ≈ 7.2 (NaOH-treated universal buffer). The plates were sandwiched together and incubated at 310 K for 4 h in a humidity-saturated atmosphere. After incubation, the sandwiched plates were separated, and next benzocaine concentrations were determined using the HPLC-DAD method. The apparent permeability coefficient (*P_app_*) was calculated using the equation:

(1) $$P_{app}=\frac{-\ln\left(1-\frac{C_{A}}{C_{equilibrium}}\right)}{S\times \left(\frac{1}{V_{D}}+\frac{1}{V_{A}}\right)\times t}$$

where: *V_D_*—donor volume, *V_A_*—acceptor volume, *C_equilibrium_*—equilibrium concentration, $C_{equilibrium}=\frac{C_{D}\times V_{D}+C_{A}\times V_{A}}{V_{D}+V_{A}}$, *S*—membrane area, and *t*—incubation time (in seconds) [17].

Compounds with *P_app_* < 1 × 10^−6^ cm s^−1^ are classified as ones of low permeability and those with *P_app_* > 1 × 10^−6^ cm s^−1^ as ones of high permeability [18,19]. The results were compared using ANOVA (Statistica 13.1, StatSoft Poland, Krakow, Poland) variance analysis. 

### 3.11. Chemical Stability Studies

For an accelerated aging test, 5.0 mg samples of the three polymorphic forms of benzocaine were weighed into 5 mL vials. To evaluate their stability at an increased air humidity, the open vials were placed in heat chambers at 353 K, in desiccators containing saturated solutions of inorganic salts: sodium bromide (≈50% RH), sodium nitrate (66% RH), sodium chloride (≈76% RH) and zinc sulfate (≈90% RH). To evaluate the stability of the benzocaine samples in dry air, the vials were immersed in a sand bath placed in the heat chambers at 383 K. At specified time intervals, determined by the rate of degradation, the vials were removed, cooled to room temperature and their contents were dissolved in a mixture of methanol:water (50:50 *v*/*v*) and injected into the chromatographic column at a concentration 0.2 mg mL^−1^. The accelerated aging test continued for 6 months.

## 4. Conclusions

The application of widely used procedures for processing APIs during preformulation and formulation work allows for obtaining three polymorphic forms of benzocaine, as confirmed by changes in PXRD diffractograms and FT-IR spectra. The presence of the polymorphic forms of benzocaine affects its physicochemical properties, such as solubility and apparent solubility. The study showed that forms I and II are characterized by better solubility and permeability compared to form III (IIIa). The shapes of apparent solubility profile curves and the values of apparent solubility corresponding to the plateau curves differed significantly between the polymorphic forms of benzocaine. With respect to differences in the solubility of those forms, various values of the apparent permeability coefficient were registered during permeability studies using the PAMPA model. It was found that the chemical stability of benzocaine did not depend on its polymorphism. In the context of therapeutic safety, considerable differences in physical properties linked to the three polymorphic forms of benzocaine should be carefully considered in the process of designing preformulation and formulation work that involves the use of such forms. 

## Figures and Tables

**Table 1:** Selected characteristic bands of benzocaine.

Theory (cm^−1^)	IR (cm^−1^)	Approximate Description
485		N–H *w*
652		C–C–C *b* in benzene ring
703	703	O–C=O *b oop* + C–H *w*
767	773	O–C=O *b oop* + C–H *w*
778		breathing benzene ring + C–O–C *b* + C–N *s* + C–H *w*
830		breathing benzene ring + C–O–C *b* + C–N *s* + C–H *w*
848	849	C–H *w* at benzene ring
864		breathing benzene ring + C–O–C *b*
890		C–O *s* + breathing benzene ring
1138	1110	C–O *s* + breathing benzene ring + C–H *r*
1200	1173	C–H *sc*
1306	1280	C–O *s* + C–C *s* + C–H *w*
1340	1312	C–H *r* + C–N *s*
1408	1368	C–H *r*
1431	1390	C–H *r*
1500	1444	C–H *sc*
1522		C–H *sc*
1560	1513	C–N *s* + C–C *s* + C–C–H *b*
1610	1596	C–N *s* + C–C *s* + C–C–H *b*
1677	1596	C=C *s* + N–H *sc* + C–C *s* + C–N *s*
1785	1682	C=O *s*
3053		C–H *s* in CH_3_ and CH_2_
3225		C–H *s* in benzene ring
3585	3341	N–H *s* sym.
3697	3425	N–H *s* asym.

*s*—stretching, *b*—bending, *r*—rocking, *w*—wagging, *sc*—scissoring, *t*—twisting, *oop*—out of the plane, sym—symmetric, asym—asymmetric.

**Table 2:** Validation parameters of the HPLC-DAD method for determination of benzocaine in methanol.

Parameter	Results			
Selectivity	Peak symmetry factor (in the range 0.8–1.5 required) = 0.94 Absence of interfering substances confirmed			
Limit of quantification (LOQ)	LOQ = 0.32 μg mL^−1^ Where SD is the average of standard deviations of determinations in the lower range of linearity and a is the directional coefficient of the plotted linear function			
Limit of detection (LOD)	LOD = 0.10 μg mL^−1^			
The range of linearity	0.35–1.50 μg mL^−1^			
Linearity	y = ax + b a = 464.76 ± 10.98 b = 214.24 ± 9.98 Correlation coefficient (r) = 0.9995			
Accuracy	Concentration	0.53 mg mL^−1^	0.67 mg mL^−1^	0.8 mg mL^−1^
	Average of three injections	0.54 mg mL^−1^	0.69 mg mL^−1^	0.86 mg mL^−1^
	SD	0.04	0.07	0.04
	RSD (<5% required)	0.45	0.71	0.35
	Recovery (95–105% required)	98.36	99.24	96.19

**Table 3:** Validation parameters of the HPLC-DAD method for the determination of benzocaine in a phosphate buffer of pH ≈ 7.2.

Parameter	Results			
Selectivity	Peak symmetry factor (in the range 0.8–1.5 required) = 1.29 Absence of interfering substances confirmed			
Limit of quantification (LOQ)	LOQ = 4.70 μg mL^−1^ Where SD is the average of standard deviations of determinations in the lower range of linearity and a is the directional coefficient of the plotted linear function			
Limit of detection (LOD)	LOD = 1.50 μg mL^−1^			
The range of linearity	5.0–20.0 μg mL^−1^			
Linearity	y = ax + b a = 955.65 ± 35.22 b = −0.50 ± 0.13 Correlation coefficient (r) = 0.9987			
Accuracy	Concentration	8.0 μg mL^−1^	10.0 μg mL^−1^	12.0 μg mL^−1^
	Average of six injections	7.9 μg mL^−1^	10.6 μg mL^−1^	13.1 μg mL^−1^
	SD	0.04	0.06	0.06
	RSD (<5% required)	0.45	0.53	0.46
	Recovery (95–105% required)	95.49	96.24	95.87

**Table 4:** Solubility of polymorphic forms of benzocaine.

Samples of Benzocaine	III	I	II	IIIa
Solubility in methanol at ambient temperature (mg mL^−1^)	46.64 ± 0.11	50.58 ± 0.15	54.65 ± 0.34	47.34 ± 0.01
Solubility in phosphate buffer (pH ≈ 7.2) at 37 °C (mg mL^−1^)	0.61 ± 0.01	0.65 ± 0.01	0.64 ± 0.01	0.62 ± 0.01

## Abbreviations

API	active pharmaceutical ingredient
I polymorphic form	benzocaine subjected to ball milling
II polymorphic form	benzocaine subjected to micro milling
III and IIIa polymorphic forms	benzocaine in initial form and form subjected to cryogenic grinding
EXP	excipient
DFT	density functional theory
FT-IR	Fourier transform infrared spectroscopy
HPLC-DAD	high-performance liquid chromatography with diode array detector
RH	relative humidity
T	temperature
PAMPA	parallel artificial membrane permeability assay
PXRD	X-ray powder diffraction
